# Supplementary material for: Magnetic Yoking and Tunable Interactions in FePt-Based Hard/Soft Bilayers
Source: Sci Rep. 2016 Sep 8;6:32842. doi: 10.1038/srep32842 (PMC5015099; doi:10.1038/srep32842)
Supplement: Supplementary Information [file srep32842-s1.doc]

**Magnetic Yoking and Tunable Interactions in FePt-Based Hard/Soft Bilayers**

Dustin A. Gilbert,^1,2^ Jung-Wei Liao,^3^ Brian J. Kirby,^2^ Michael Winklhofer,^4,5,6^

Chih-Huang Lai,^3^ and Kai Liu^1,*^

*^1^Dept. of Physics, University of California, Davis, California 95616, USA*

*^2^NIST Center for Neutron Research, Gaithersburg, Maryland 20899, USA*

*^3^Dept. of Materials Science and Engineering, National Tsing Hua University, Hsinchu 30013, Taiwan*

*^4^Dept. of Earth and Environmental Sciences, Geophysics, Munich University, 80333 Germany*

*^5^Faculty of Physics, University of Duisburg-Essen, 47057 Duisburg, Germany*

*^6^IBU, School of Mathematics and Science, Carl von Ossietzky University, 26129, Oldenburg, Germany*

**Supplementary Information**

Similar experiments were performed on *L*1_0_-FePt/Fe bilayer films. The major loop behavior, Supplementary Fig. S1, is similar to those with *A*1-FePt films featured in the main text. Comparing the relative changes of the *A*1-FePt and Fe soft layers, the Fe reduces the bilayer coercivity much more significantly (from 340 mT to 165 mT for 2nm Fe vs. 252 mT for 2 nm *A*1-FePt) than the *A*1-FePt. The FORC distributions for these films are shown in Supplementary Fig. S2. Similar to the distributions in the main text, the FORCs consist of a vertical and horizontal ridge features. Here, even the thinnest Fe layer causes the FORC distribution to shift to a 'T' configuration, and evolve continuously to a right bending construction with thicker Fe. *Δ*M measurements, shown in Supplementary Fig. S3, confirm that for all thicknesses of Fe the dipolar interactions are dominant. For the Fe soft layer the dipolar interactions are enhanced more than in the *A*1-FePt case likely due to an enhanced yoking effect from the higher *M_S_* in Fe (1700 emu·cm^-3^, 1 emu = 10^-3^ A·m^2^) compared to *A*1-FePt (1200 emu·cm^-3^). An increased *M_S_* also favors an in-plane magnetization at thinner soft-layer thicknesses due to the gain in magnetostatic energy.


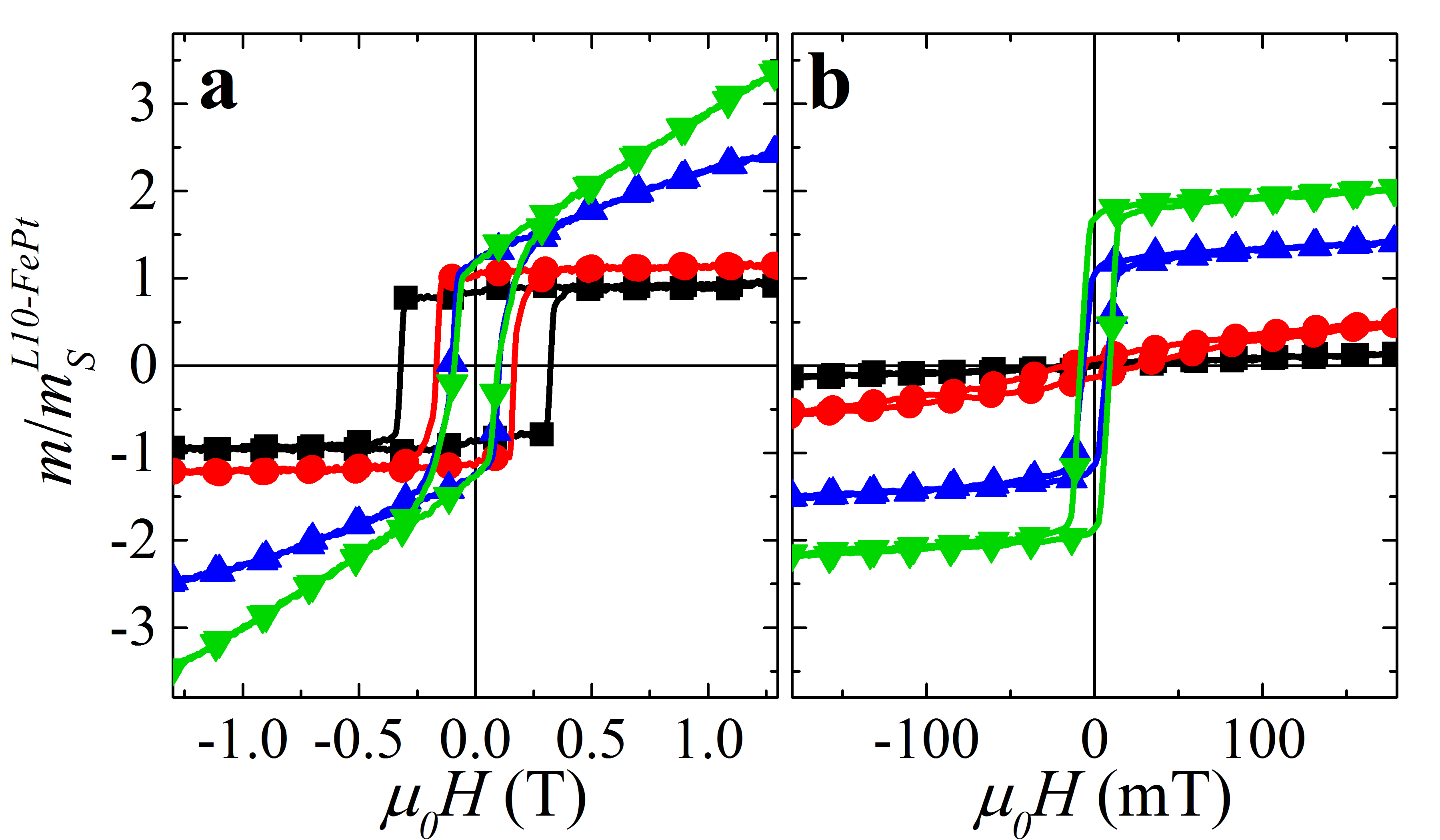


**Supplementary Fig. S1. Magnetometry**. Major Hysteresis Loops of *L*1_0_-FePt (4 nm) / Fe (*t_Fe_*) films with the magnetic field applied in the (**a**) perpendicular and (**b**) in-plane orientation. Samples are identified by color and symbol for *t_Fe_*=0 nm (black squares), *t_Fe_*=3 nm (red circles), *t_Fe_*=5 nm (blue triangles), and *t_Fe_*=9 nm (green inverted triangles).


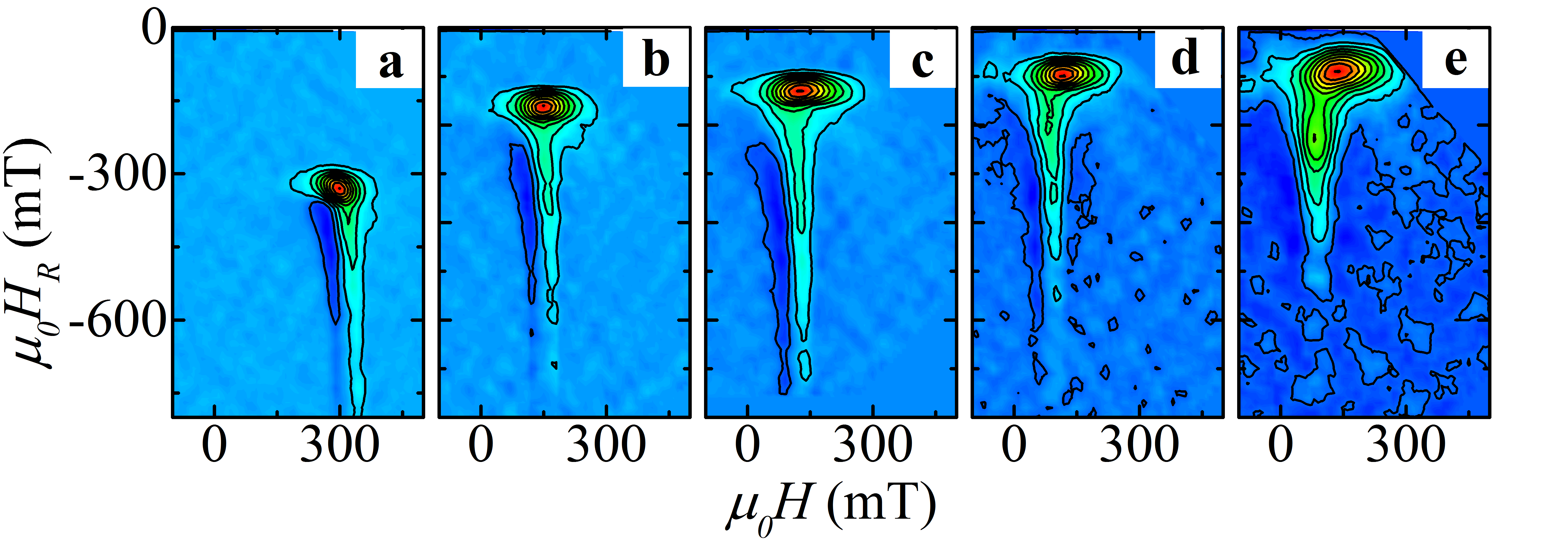


**Supplementary Fig. S2. FORC distributions.** FORC distributions of *L*1_0_-FePt/Fe(*t_Fe_*) films where *t_Fe_* is (**a**) 0 nm, (**b**) 2 nm, (**c**) 3 nm, (**d**) 5 nm, and (**e**) 9 nm.


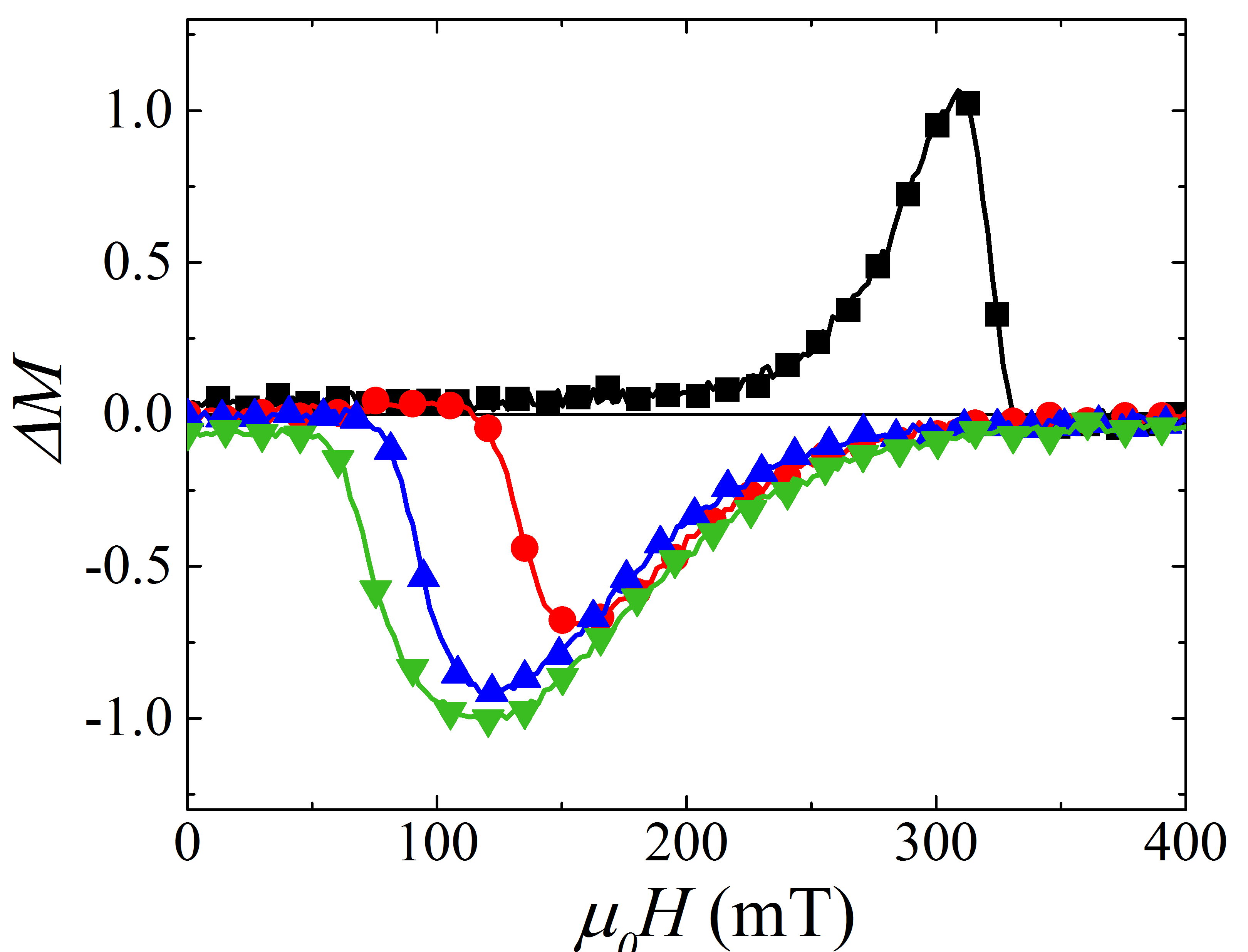


**Supplementary Fig. S3. *ΔM* measurements.** *ΔM* plot for *L*1_0_-FePt (4 nm) /Fe(*t_Fe_*) films. Samples are identified by color and symbol for *t_Fe_*=0 nm (black squares), *t_Fe_*=3 nm (red circles), *t_Fe_*=5 nm (blue triangles), and *t_Fe_*=9 nm (green inverted triangles).

To estimate the strength of the dipolar field in the *L*1_0_-FePt/*A*1-FePt system, we have modeled the system as 280 nm sized square domains with opposite out-of-plane orientations (found in bare *L*1_0_‑FePt film) with a 5 nm non-contributing domain wall region. The in-plane dipolar field from these domains was calculated at 4 nm above the *L*1_0_-FePt film. The net in-plane dipolar field is found to be greater than 100 mT over the domain wall, and greater than 4 mT - the *A*1-FePt coercivity - up to 50 nm (laterally) from the domain boundary. Thus, it is reasonable that the soft layer orientation indeed follows the dipolar fields (yoking) and as a result acts to moderate interactions between domains in the hard layer.
